# Characterization and identification of a novel chromosomal class C β-lactamase, LAQ-1, and comparative genomic analysis of a multidrug resistance plasmid in *Lelliottia amnigena* P13

**DOI:** 10.3389/fmicb.2022.990736

**Published:** 2022-11-23

**Authors:** Anqi Li, Chunxia Yan, Lei Zhang, Shuang Liu, Chunlin Feng, Linhua Zhang, Fubo Dong, Xiusheng Sheng, Lan Wang, Yanfang Zhang, Junwan Lu, Jiefeng Xu, Lin Zheng, Qiyu Bao, Cong Cheng, Dawei Huang

**Affiliations:** ^1^School of Medicine, Lishui University, Lishui, China; ^2^Key Laboratory of Medical Genetics of Zhejiang Province, Key Laboratory of Laboratory Medicine, Ministry of Education of China, School of Laboratory Medicine and Life Sciences, Wenzhou Medical University, Wenzhou, China; ^3^Medical Molecular Biology Laboratory, School of Medicine, Jinhua Polytechnic, Jinhua, China; ^4^The People’s Hospital of Yuhuan, Yuhuan, China; ^5^Department of Clinical Laboratory, Hospital of Ningbo University School of Medicine, Ningbo, Zhejiang, China

**Keywords:** *Lelliottia amnigena* P13, novel resistance gene, β-lactamase, *bla*_LAQ-1_, kinetic parameter

## Abstract

**Introduction:**

*Lelliottia amnigena*, a bacterium usually isolated from natural environments, may cause human infections and has been suggested to be naturally resistant to second- and third-generation cephalosporins.

**Methods:**

In this study, we determined the whole-genome sequence of an isolate, *L. Amnigena* P13, isolated from animal farm sewage. On the basis of genome sequence analysis, susceptibility testing, molecular cloning, and enzyme kinetic parameter analysis, we identified a novel chromosome-encoded AmpC β-lactamase, LAQ-1.

**Results and Discussion:**

*bla*_LAQ-1_ is resistant to penicillin G, ampicillin, and several first- to fourth-generation cephalosporins, such as cefazolin, cefoxitin and cefepime. The MIC levels of some β-lactams, such as cefoxitin, cefepime, aztreonam and cefazolin, for the recombinant clone (pUCP24-*bla*_LAQ-1_/DH5α) increased by approximately 4- to 64-fold compared with those of the control strain (pUCP24/DH5α). The kinetic properties of LAQ-1, with the highest catalytic activity observed toward piperacillin, were basically the same as those of typical class C β-lactamases, and avibactam had a strong inhibitory effect on its hydrolytic activity. The genetic background of *bla*_LAQ-1_ was relatively conserved, and no mobile genetic element (MGE) was found around it. The plasmid pP13-67 of L. amnigena P13 harbored 12 resistance genes [*qnrS1, aph(6)-Id, aadA2, sul1, sul2,bla*_TEM-1_, qacEΔ1, *dfrA12*, *tetA* and *floR*] related to different mobile genetic elements within an ~22 kb multidrug resistance region. The multidrug resistance region shared the highest nucleotide sequence similarities with those of the chromosomes or plasmids of different bacterial species, indicating the possibility of horizontal transfer of these resistance genes among different bacterial species.

## Introduction

In 1981, Enterobacter H3 was renamed *Enterobacter amnigenus* based on molecular hybridization (Izard et al., 1981). It was then proposed that the bacterium could be divided into two groups based on the molecular hybridization data (Izard et al., 1981). Subsequently, according to biochemical experiments, *E. amnigenus* was classified into two biogroups (Casin et al., 1986): Strains in Biogroup 1 ferment sucrose and raffinose but not D-sorbitol. In contrast, those in Biogroup 2 ferment D-sorbitol but not sucrose or raffinose (Rd et al., 1985). In 2013, *E. amnigenus* and *E. nimipressuralis* were reclassified as belonging to a new genus of the family *Enterobacteriaceae* designated *Lelliottia* based on multilocus sequence typing (MLST), DNA molecular hybridization, phenotypic sugar fermentation characteristics and cell wall fatty acid spectrum analysis (Brady et al., 2013). *E. amnigenus* was renamed *Lelliottia amnigena* and *E. nimipressuralis* was reclassified as *Lelliottia nimipressuralis.* Members of the genus *Lelliottia* are rod-shaped, motile and facultative anaerobic Gram-negative bacilli (Liu and Tang, 2016). *L. amnigena* was suspected to be pathogenic in humans since it was first isolated in nature, which was confirmed when it was isolated in samples from at least four patients whose clinical laboratory results indicated (Martin Guerra et al., 2018). *Lelliottia amnigena* can be isolated from a variety of environmental sources (e.g., soil, river/lake; Rd et al., 1985), food (e.g., milk, mushrooms, artisanal raw cheeses) (Yuk et al., 2018) and human clinical samples (e.g., respiratory tract, wound, blood, and stool samples). Moreover, it is an uncommon/unusual pathogen capable of causing sporadic reports of pyonephrosis, urinary tract infection, sepsis and endophthalmitis (Bollet et al., 1991; Westerfeld et al., 2009; Leal-Negredo et al., 2017). The natural resistance of *L. amnigena* to second- and third-generation cephalosporins such as cefoxitin, cefotaxime and cefaclor was described in the most extensive study of susceptibility (Murugaiyan et al., 2015). The research also showed that some strains of *L. amnigena* were resistant to gentamicin, doxycycline, nitrofurantoin and β-lactam/β-lactamase inhibitor combinations (such as amoxicillin/clavulanic; Fadare and Okoh, 2021). However, contradictory result had been reported that no natural resistance to cephalosporins, including cefoxitin, yet a decreased susceptibility to cefixime, cefpodoxime and ceftibutene was found (Stock and Wiedemann, 2002). To date, several studies have reported that *L. amnigena* possesses the *ampC* gene. However, no detailed studies on the *L. amnigena* AmpC β-lactamase have been documented (Literak et al., 2014).

β-lactamase is an enzyme that can hydrolyze the β-lactam ring and inactivate antibiotics before they bind to penicillin-binding proteins (Knotthunziker et al., 1982). AmpC β-lactamase (AmpC) is a serine-active protease and one of the important β-lactamases. In the Ambler molecular structure classification, it belongs to class C (Bush et al., 1995), with an apparent molecular weight of 38 ~ 42 kDa, and is distributed mainly in periplasm (Medeiros, 1989). Overexpression of the chromosomally encoded AmpC enzyme is the main mechanism underlying the resistance of Gram-negative bacilli to penicillin, cephalosporin, cephamycin and monocyclic β-lactam antibiotics (Jacoby, 2009). At the same time, the AmpC enzyme encoded by chromosomal and plasmid genes is also evolving (Hanson, 2003). Overexpression of this enzyme confers resistance to a wide range of cephalosporins, including cefotaxime, ceftazidime and ceftriaxone. Some organisms even show mutations that reduce influx (loss of outer membrane porin) or enhance efflux (activation of efflux pump; Hanson, 2002). Common β-lactamase inhibitors such as clavulanic acid, sulbactam and tazobactam had no obvious inhibitory effect on the AmpC enzyme, but avibactam had an inhibitory effect on the AmpC enzyme (Bush et al., 1993). Although the AmpC enzyme causes less resistance than extended-spectrum β-lactamases (ESBLs) in most parts of the world, it is more difficult to detect and has a wider range. Therefore, more in-depth research on AmpC will be of great significance for guiding clinical testing and follow-up medication.

In this study, we determined the whole-genome sequence of an isolate, *L. amnigena* P13, isolated from animal farm sewage. On the basis of genome sequence analysis, susceptibility testing, molecular cloning, and enzyme kinetic parameter analysis, we identified a novel chromosome-encoded AmpC β-lactamase, LAQ-1.

## Materials and methods

### Bacterial strains

*Lelliottia amnigena* P13 was isolated from sewage discharged from an animal farm in Wenzhou, Zhejiang Province, China. The strain was identified by a bioMérieux VITEK 2 compact instrument (bioMérieux, Marcy l’Etoile, France) and then verified by 16S rRNA gene homology and average nucleotide identity (ANI) analyses. The bacteria used in this work are listed in Table 1.

**Table 1:** Bacteria and plasmids used in this work.

Strain or plasmid	Relevant characteristic(s)	Reference or source
P13	The original strain of *L. amnigena* P13	This study
DH5α	*Escherichia coli* DH5α was used as a host for cloning of the *bla*_LAQ-1_ gene	Our laboratory collection
BL21	*E. coli* BL21 was used as a host for expression of LAQ-1	Our laboratory collection
ATCC 25922	*E. coli* ATCC 25922 was used as a quality control for antimicrobial susceptibility testing	Our laboratory collection
pUCP24-*bla*_LAQ-1_*/*DH5α	DH5α carrying the recombinant plasmid pUCP24-*bla*_LAQ-1_	This study
pET-28a-*bla*_LAQ-1_/BL21	BL21 carrying the recombinant plasmid pET-28a-*bla*_LAQ-1_	This study
pUCP24	Cloning vector for the PCR products of the *bla*_LAQ-1_ gene with its upstream promoter region, GEN^r^	Our laboratory collection
pET 28a	Expression vector for the PCR products of the ORF of the *bla*_LAQ-1_ gene, KAN^r^	Our laboratory collection

^r^Resistance; GEN, gentamicin; KAN, kanamycin.

### Antimicrobial susceptibility testing

According to the guidelines of the Clinical and Laboratory Standards Institute (CLSI), the minimum inhibitory concentrations (MICs) were determined by the agar dilution method. Drug susceptibility was determined according to the CLSI drug susceptibility test standard (CLSI, 2021) and the guidelines of the European *Enterobacteriaceae* Antimicrobial Susceptibility Test Committee (EUCAST, 2019). *E. coli* ATCC 25922 was used as the reference strain for quality control.

### Whole-genome sequencing and functional annotation of the genome sequence

The whole-genome DNA of *L. amnigena* P13 was extracted with the AxyPrep Bacterial Genomic DNA Miniprep Kit (Axygen Science, Union City, CA, United States) and sequenced with the Illumina HiSeq 2,500 (Shanghai, China) and PacBio RS II (Pacific Biosciences) platforms. The PacBio long reads were initially assembled by Canu (Koren et al., 2017), and then two FASTQ sequence files generated by the Illumina sequencer were mapped onto the primary assembly to control assembly quality and to correct possibly misidentified bases using Pilon (Walker et al., 2014). Prokka was used to predict the potential open reading frames (ORFs; Seemann, 2014) of the assembled genome, and the functional annotation of these proteins was performed by DIAMOND (Buchfink et al., 2021) with an e-value threshold of 1e-5 against the nonredundant protein sequence (NR) database of the National Center for Biotechnology Information (NCBI). The drug resistance genes were annotated by drug resistance gene recognizer (RGI) software against the comprehensive antibiotic resistance database (Mcarthur et al., 2013). The mobile genetic elements (MGEs) were annotated using ISfinder (Siguier et al., 2006) and INTEGRALL (Siguier et al., 2006). ANI was calculated using FastANI (Jain et al., 2018). The basic characteristics of the plasmid and the comparison with its close relatives were visualized by the CGView Comparison Tool (Petkau et al., 2010). The molecular weight and the pI value of LAQ-1 were predicted using ProtParam^1^. The putative signal peptide cleavage site of LAQ-1 was identified by SignalP 5.0 (Almagro Armenteros et al., 2019). Multiple sequence alignment of the amino acid sequences, neighbor-joining phylogenetic tree construction and visualization of LAQ-1 with other AmpC β-lactamases were performed using MAFFT (Kazutaka and Standley, 2013), MEGA (Kumar and Tamura, 2016) and ggtree (Yu, 2020), respectively. The structures of the approximately 20-kb flanking regions of the *bla*_LAQ-1_ gene and its relatives were compared using the GenePlotR package (Guy et al., 2010). GenePlotR was also used to generate graphs showing the structural comparison and nucleotide identity of different MDR regions (Sullivan et al., 2011). GNU Parallel was used to access various data in the NCBI database in parallel.

### Cloning of the *bla*_LAQ-1_ gene and expression and purification of LAQ-1

The primers to amplify the *bla*_LAQ-1_ gene together with its promoter region (pro-*bla*_LAQ-1_) were designed using SnapGene Viewer (Qiao et al., 2019), with a pair of restriction endonuclease digestion sites (*Bam*HI and *Hind*III) included at the 5′-end of both primers. The forward primer sequence was 5’-CGCGGATCCGCGTGCAAAATCCGGTGGTGGTGATC-3′, and the reverse primer sequence was 5’-CCCAAGCTTGGGATCGGTTATTTCAACGTGTCCAGGA-3′ (Supplementary Table S1). Then, the PCR product was eluted from an agarose gel and digested with *Bam*HI and *Hind*III, and the digested product was ligated into the pUCP24 vector, which was also digested with *Bam*HI and *Hind*III, by a T4 DNA Ligase Cloning Kit (Takara Bio, Inc., Dalian, China). The recombinant plasmid (pUCP24-pro-*bla*_LAQ-1_) was transformed into competent *E. coli* DH5α cells by the calcium chloride method (Cohen et al., 1972), and the transformants (pUCP24-pro-*bla*_LAQ-1_/DH5α) were cultured on Luria-Bertani (LB) agar plates containing 40 μg/mL gentamicin. The cloned resistance gene in the recombinant plasmid was verified by Sanger sequencing (Shanghai Sunshine Biotechnology Co., Ltd., Shanghai, China). The resistance activity of the cloned gene *bla*_LAQ-1_ was further determined. To obtain the β-lactamase LAQ-1, the ORF of *bla*_LAQ-1_ without the signal sequence was cloned by the above mentioned procedure, using pET-28a as the cloning vector and *E. coli* BL21 as the recipient. The recombinant clone (pET-28a-*bla*_LAQ-1_/BL21) was selected on LB agar plates containing 50 μg/mL kanamycin. The recombinant clone was cultured overnight in LB medium containing 50 μg/mL kanamycin. The overnight culture was diluted 100-fold in 100 mL of LB medium and shaken at 37°C for 2–3 h until the OD_600_ reached 0.6–0.8. Isopropyl-β-d-thiogalactopyranoside (IPTG; Sigma Chemicals Co., St. Louis, MO, United States) was added at a final concentration of 1 mM, and incubation was continued for an additional 4 h. According to the instructions for BeyoGold His tag Purification Resin (Beyotime, Shanghai, China), the protein was purified by affinity chromatography, followed by incubation with Enterokinase (GenScript, Nanjing, China) at 16°C for 36 h to remove the histidine tag (Zhou et al., 2020).

### Determination of kinetic parameters

Kinetic parameters for hydrolysis of β-lactams by the purified LAQ-1 β-lactamase were examined using a UV–VIS spectrophotometer (U-3900, HITACHI, Japan) at 37°C in 10 mM phosphate buffer (pH 7.0) in a final reaction volume of 200 μl. The steady-state kinetic parameters (*k_cat_* and *K_m_*) were determined by nonlinear regression of the initial reaction rates with the Michaelis–Menten equation in GraphPad Prism 8 (GraphPad Software, CA, United States). The inhibitory effect of β-lactamase was studied using 100 μM nitrocephalosporin as a substrate (Chen et al., 2019). The β-lactamase inhibitor avibactam and GMP (disodium 50-guanosine monophosphate) were preincubated with purified LAQ-1 at different concentrations for 5 min at 37°C. The inhibitor concentration required to reduce the hydrolysis of 100 μM nitrocefin by 50% was determined by nonlinear regression with the log (inhibitor) versus response-(three parameters) in GraphPad Prism 8 (Chen et al., 2019).

### Nucleotide sequence accession numbers

The chromosome, the plasmid pP13-67 and the *bla*_LAQ-1_ gene sequences of *L. amnigena* P13 have been submitted to GenBank with accession numbers CP099511.1, CP099512.1 and MZ497396.1, respectively.

## Results and discussion

### Genome characteristics and resistance profile of *Lelliottia amnigena* P13

*Lelliottia amnigena* P13 was isolated from sewage discharged from an animal farm in Wenzhou, southern China, in 2017. The 16S rRNA gene sequence homology analysis showed that P13 was most closely related to *L. amnigena* NCTC12124 (with a coverage of 98% and an identity of 99.93%). The genome-wide ANI analysis between the genomes of P13 and other bacteria in the NCBI assembly database revealed that the 20 bacterial genomes showed ≥98.00% ANI with that of P13 were all from *L. amnigena*, of which the genome of the type strain *L. amnigena* NCTC 12124 (GenBank: LR134135.1) showed 98.84% ANI with the P13 genome. It confirmed that the isolate belonged to the species *L. amnigena* and thus designated *L. amnigena* P13.

**Figure 1:**
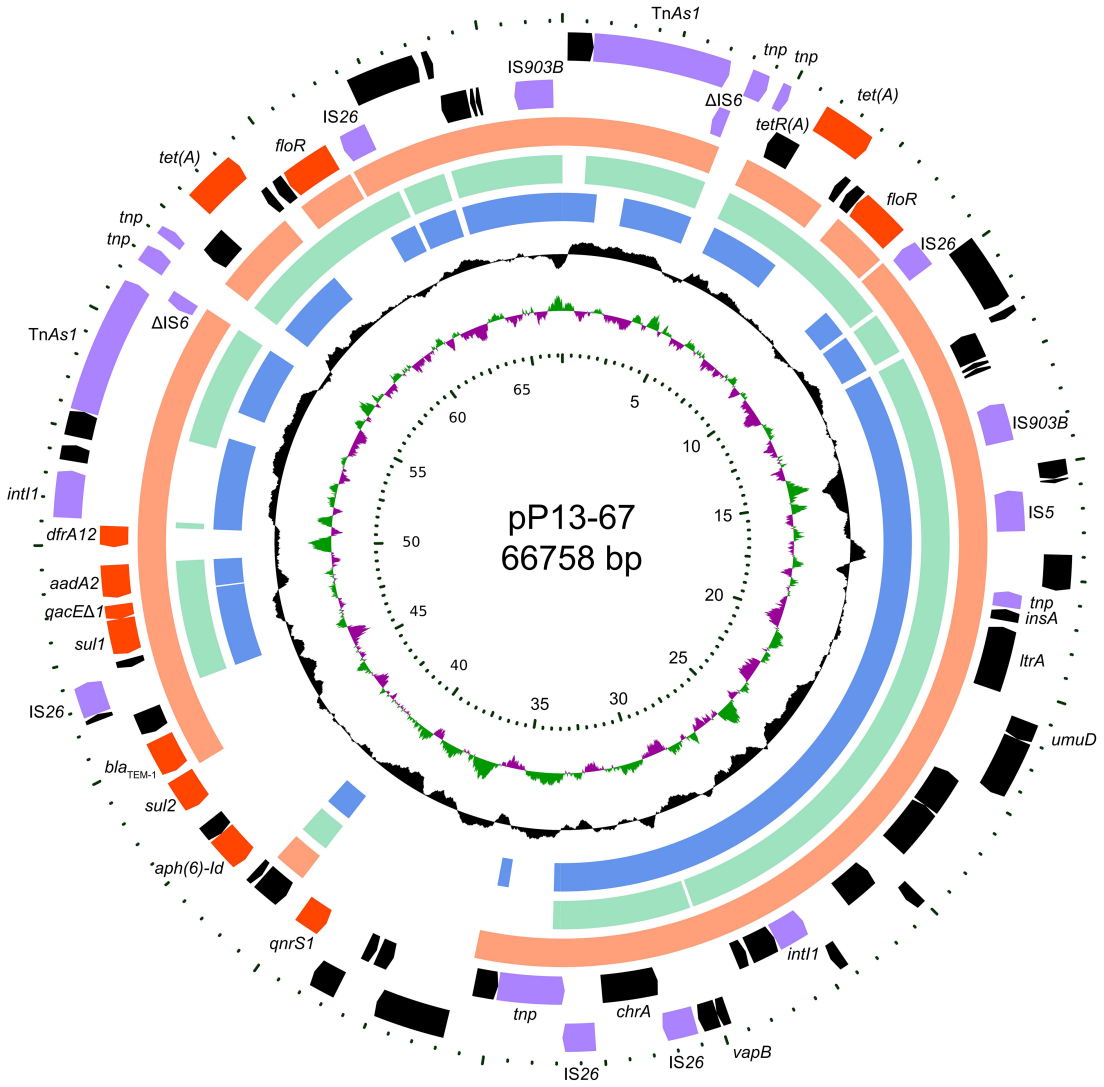
Circular map of pP13-67 and comparative genomic analysis with other closely related plasmids. From inside to outside: Circle 1 shows the scale in kb. Circles 2 and 3 display the GC skew and GC content, respectively. Circles 4–6 show the homologous sequences of pEcl5-2 of *Enterobacter hormaechei* Eho-5 (CP047738.1), pN56639 of *Escherichia coli* CVM N56639 (CP043753.1) and p1 of *Klebsiella pneumoniae* JM45 (CP006657.1), respectively, while areas displaying different features have been left blank. Circles 7 and 8 present genes encoded in the reverse and forward strands of pP13-67, respectively.

The P13 genome consists of a circular chromosome and a plasmid designated pP13-67. The chromosome size is 4,555,627 bp with a GC content of 52.85%, and it encodes 4,349 ORFs (Table 2). pP13-67 was 66,758 bp in length and encoded 79 ORFs including 11 resistance genes related to eight classes of antimicrobials (Figure 1). Similar to most *Enterobacteriaceae*, the *in vitro* drug susceptibility test showed that *L. amnigena* P13 was resistant to ampicillin, penicillin G, cefoxitin, cefazolin, tetracycline and ticarcillin/clavulanate (Table 3).

**Table 2:** General features of the *L.amnigena* P13 genome.

	Chromosome	pP13-67
Size (bp)	4,555,627	66,758
GC content (%)	52.85	56.08
Predicted coding sequences (CDSs)	4,240	79
Known proteins	3,951	65
Hypothetical proteins	289	14
Protein coding (%)	97.49	100
Average ORF length (bp)	931	690
Average protein length (aa)	314	229
tRNAs	86	0
rRNA operons	(16S-23S-5S)*7, 5S	0

**Table 3:** MICs of 19 antimicrobials for the recombinants and the control strains (μg/mL).

	ATCC25922	DH5α	pUCP24/DH5α	pUCP24-*bla*_LAQ-1_/DH5α	P13
Ampicillin	4	2	4	64	256
penicillin G	32	16	16	4	>1,024
cefoxitin	2	2	2	128	256
cefazolin sodium	2	2	2	8	8
ceftazidime	0.25	0.25	0.125	1	0.5
imipenem	0.125	0.125	0.5	0.25	0.25
cefepime	0.06	0.06	<0.03	0.125	0.06
aztreonam	0.25	0.125	0.25	1	0.06
ticarcillin	4	2	1	8	32
piperacillin	2	2	1	4	16
cloxacillin	256	256	256	512	1,024
nalidixic acid	4	8			16
chloramphenicol	4	8			8
tetracycline	4	2			64
Amp/Sulbactam	2	2			2
Ticarcillin/Clavulanate	8	4	2	8	16
Piperacillin/Tazobactam	2	2	2	4	8
Amikacin	4	4			<0.5
Gentamicin	0.25	<0.125			<0.125

### Identification of the novel β-lactamase gene *bla*_LAQ-1_

When analyzing the resistance mechanism, we found that only one of the function- characterized resistance gene for β-lactam antibiotics (a *bla*_TEM-1_ encoded on the plasmid pP13-67) was annotated in the P13 genome, even though this strain shows resistance to many kinds of β-lactam antibiotics tested. To investigate whether there was a putative β-lactamase gene encoded in the *L. amnigena* P13 genome, we checked the annotation result of the genome and found that one of the predicted genes contained the conserved motif of an Ambler class C β-lactamase, which showed the highest amino acid homology (with an identity of 78.95%) with the function-characterized AmpC β-lactamase ACT-22 (Porres-Osante et al., 2015). Other β-lactamases with higher amino acid identities to LAQ-1 were ACT-6 (ACJ05686.1, 78.42%, 298/380), CMY-20 (AAX58682.2, 78.42%, 298/380) and so on.

To determine the potential resistance function of the gene, we cloned the ORF of the predicted gene (finally designated *bla*_LAQ-1_) with its promoter region into the pUCP24 vector. The *bla*_LAQ-1_ gene was functional, and the recombinant clone (pUCP24-*bla*_LAQ-1_/DH5α) showed increased MIC levels (by approximately 4- to 64-fold) for some β-lactams, including several first- to fourth-generation cephalosporins, such as cefazolin, cefoxitin and cefepime, compared with the control strains (DH5α or DH5α carrying pUCP24; Table 3). The resistance profile of *bla*_LAQ-1_ is consistent with that of most *Enterobacter* species with inducible *ampC* genes (Jacoby, 2009). However, the recombinant clone harboring *bla*_LAQ-1_ did not show any change in MIC levels to carbapenems. *bla*_LAQ-1_ conferred resistance to fourth-generation cephalosporins, unlike ACT-6 (Zhu et al., 2011) and CMY-2 (Bauernfeind et al., 1996). Classic class A β-lactam inhibitors, such as clavulanic acid and sulbactam, had poor inhibitory effects on the activity of LAQ-1 (Table 3).

### Molecular characterization of the novel AmpC β-lactamase LAQ-1

The novel β-lactamase gene *bla*_LAQ-1_, which is 1,143 bp in length, encodes a 380 amino acid preprotein of approximately 39.6 kDa. The cleavage site of the signal peptide is predicted to be located between amino acid residues 19 (alanine) and 20 (alanine). It is further predicted that the isoelectric point of LAQ-1 is 8.58. Although a putative class C β-lactamase (LR134135.1) showing the highest nucleotide homology with *bla*_LAQ-1_ (99.39%, 1,136/1,143) was found to be encoded in the *L. amnigena* NCTC12124 chromosome (NZ_CP023529.1), the two function-characterized β-lactamases with the highest amino acid identities to LAQ-1 were ACT-6 (ACJ05686.1, 78.42%, 298/380) and CMY-20 (AAX58682.2, 78.42%, 298/380).

**Figure 2:**
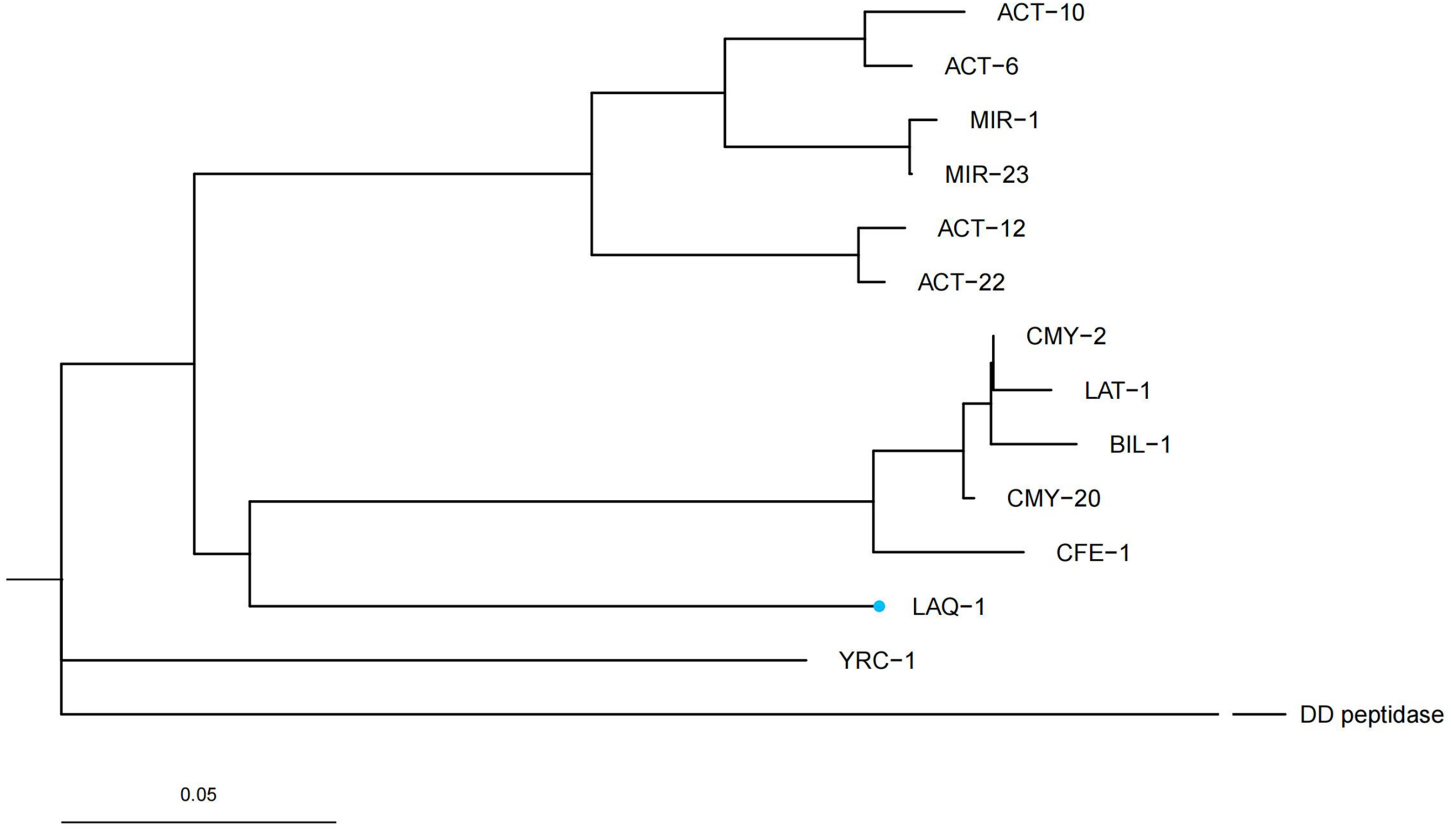
A rooted phylogenetic tree of LAQ-1 with its function-characterized relatives. DD peptidase of *Streptomyces* R61 (D-alanyl-d-alanine-Carboxypeptidase, 1CEF_A) was used as an out group. LAQ-1 from our study is shown with a blue dot. The other sequences and their accession numbers are ACT-12 (AFU25650.1), ACT-22 (AHM76774.1), BIL-1 (CAA52618.1), CMY-2 (CAA62957.1), CMY-20 (AAX58682.2), LAT-1 (CAA55007.1), CFE-1 (BAC76072.1), YRC-1 (ABA70720.1), MIR-1 (AAD22636.1), MIR-23 (AIG20028.1), ACT-6 (ACJ05686.1), ACT-10 (AEV91214.1).

**Figure 3:**
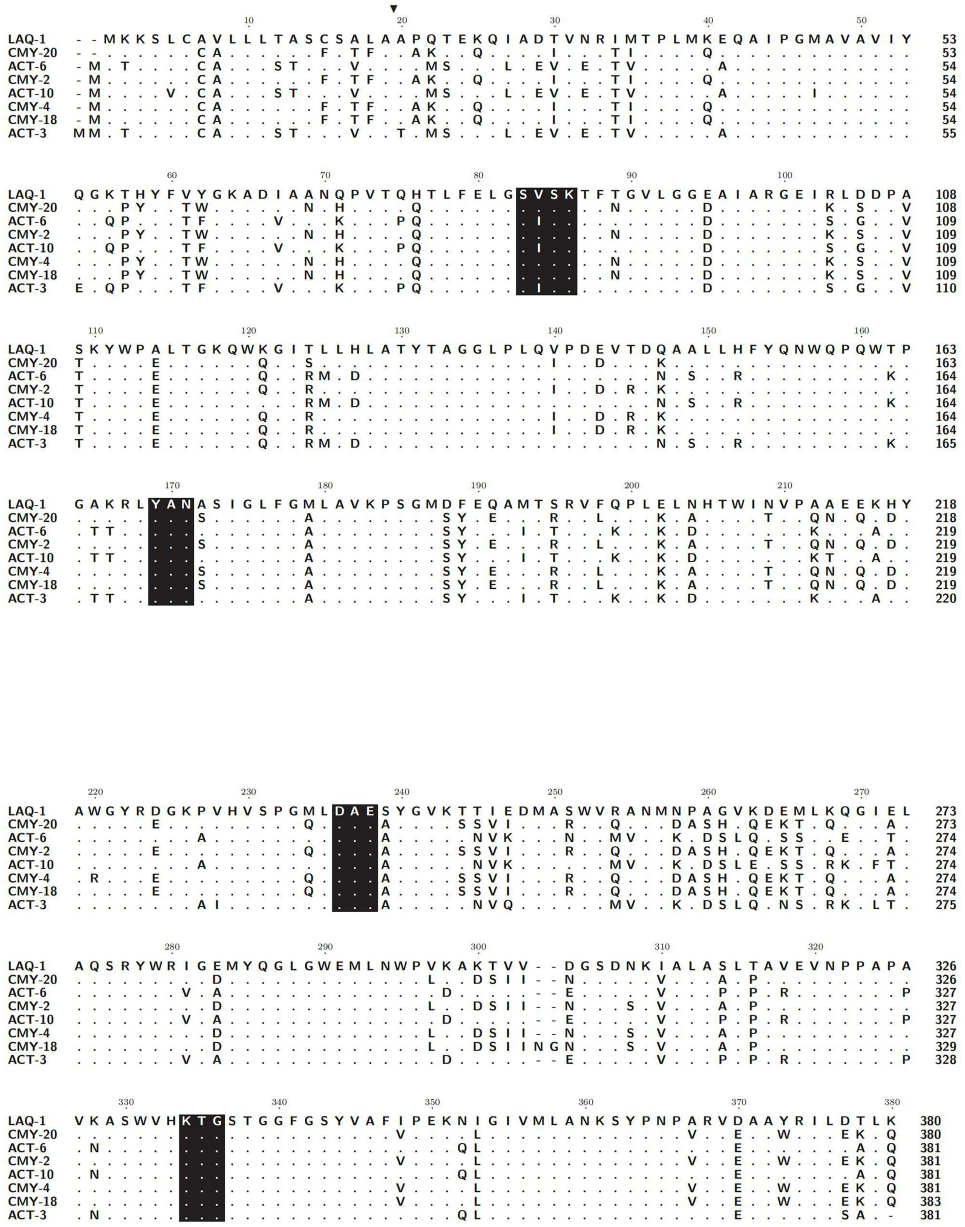
Alignment of the amino acid sequences of LAQ-1 and its homologous function-characterized class C β-lactamases. Identical amino acids are indicated by dots. Signal sequences are highlighted in black, and four conserved motifs are shaded in black. LAQ-1 numbering is based on the standard numbering scheme for class C beta-lactamases. The sequences and their accession numbers are CMY-20 (AAX58682.2), ACT-6 (ACJ05686.1), CMY-2 (CAA62957.1), ACT-10 (AEV91214.1), CMY-4 (CAA07695.1), CMY-18 (AAU95778.1) and ACT-10 (ABL67017.1).

Five genes with the highest nucleotide similarity to *bla*_LAQ-1_ (>93.79%) were retrieved from the NCBI nucleotide database, all of which originated from *L. amnigena* (one was isolated from soil, while the sources of the remaining four are unknown). The rooted phylogenetic tree analysis demonstrated that LAQ-1 formed a new branch on the phylogenetic tree of AmpC β-lactamases (Figure 2). Comparison of the deduced amino acid sequence of LAQ-1 with the function-characterized β-lactamases revealed that LAQ-1 showed the highest identities of 78.42, 78.42, 77.89, 77.89, 77.63, 77.49 and 77.11% with ACT-6, CMY-20, CMY-2, ACT-10, CMY-4, CMY-18 and ACT-10, respectively (Figure 3). Notably, all of these deduced amino acid sequences had the obligatory serine-active site of the β-lactamase catalytic motif S-X-S-K (serine-valine-serine-lysine) at positions 83–86, the typical class C β-lactamase motif Y-A-N (tryptophan-alanine-asparagine) at positions 169–171, D/E (a peptide segment containing two dicarboxylic amino acids) at positions 236–238 and the conserved triad K-T-G (lysine-threonine-glycine) at positions 334–336 (Ghuysen, 1991; Figure 3).

### Kinetic parameters of LAQ-1

The purified β-lactamase LAQ-1 showed the highest catalytic efficiency for piperacillin (*k_cat_*/*K_m_* of 107.68) and showed moderate catalytic efficiency against first- (cefazolin, *k_cat_*/*K_m_* of 39.88) and second-generation cephalosporins (cefoxitin, *k_cat_*/*K_m_* of 16.92), and penicillins (ampicillin, *k_cat_*/*K_m_* of 20.96). It demonstrated low catalytic efficiencies against the third-generation cephalosporin (such as ceftazidime with a *k_cat_*/*K_m_* of 4.58) and the extended-spectrum cephalosporins (such as cefepime, with a *k_cat_*/*K_m_* of 8.18; Table 4), which is similar to the values for other chromosome- or plasmid-encoded class C β-lactamases, such as ACT-6 (Zhu et al., 2011) and CMY-2 (Bauernfeind et al., 1996). LAQ-1 was more active against cefazolin and cefoxitin than ACT-6 (Zhu et al., 2011) because of its much higher *k_cat_*, which was partially compensated by its higher *K_m_* values (Table 4). However, the enzyme kinetic hydrolytic activities were not completely consistent with the changes in the MIC levels of the recombinant clone (pUCP24-*bla*_LAQ-1_/DH5α). For example, LAQ-1 had obvious hydrolytic activity against cefazolin (*k_cat_*/*K_m_* of 39.88) and piperacillin (*k_cat_*/*K_m_* of 191.5), but the recombinant with *bla*_LAQ-1_ showed no significant change in the MIC level (increased merely 4-fold) compared to the control strains, while the MIC of cefoxitin (*k_cat_*/*K_m_* of 16.92) changed greatly (increased 64-fold). This phenomenon may be attributed to its low activity *in vitro* (Toth et al., 2016). In contrast, no obvious hydrolytic activity of LAQ-1 against aztreonam was detected, which contradicts the increase in the MIC value of it (increased 4-fold). It has been reported that the hydrolysis rate of aztreonam is slower than that of other β-lactams, which may make its hydrolysis difficult to detect (David et al., 2012). A similar phenomenon was observed for CMY-2, the recombinant form of which had an increased MIC value against aztreonam, but the hydrolysis of aztreonam was not detectable (Kotsakis et al., 2015). The half inhibitory concentrations (IC50) of β-lactamase inhibitors showed that avibactam (IC50: 0.001451 μM) had a strong inhibitory effect on LAQ-1 and a weaker inhibitory effect on GMP (IC50: 2.626 μM). This result is consistent with the nature of AmpC β-lactamase inhibition (David et al., 2012).

**Table 4:** Kinetic parameters of the β-lactamase LAQ-1 for β-lactam antimicrobials.

Substrate	*K_m_* (μM)	*k*_cat_ (s^−1^)	*k*_cat_/*K_m_* (μM^−1^·s^−1^)
Piperacillin	87.56	9,428.44	107.68
Ampicillin	436.1	9,141.15	20.96
Cefepime	57.05	466.64	8.18
Cefoxitin	65.4	1,106.67	16.92
Cefazolin	645	25,725.41	39.88
Ceftazidime	140	641.61	4.58
Aztreonam	NH^a^	NH^a^	NH^a^
Meropenem	NH^a^	NH^a^	NH^a^
Imipenem	NH^a^	NH^a^	NH^a^

Values are the means of three independent measurements. ^a^NH, no detectable hydrolysis.

### Genetic context of the *bla*_LAQ-1_ gene

**Figure 4:**
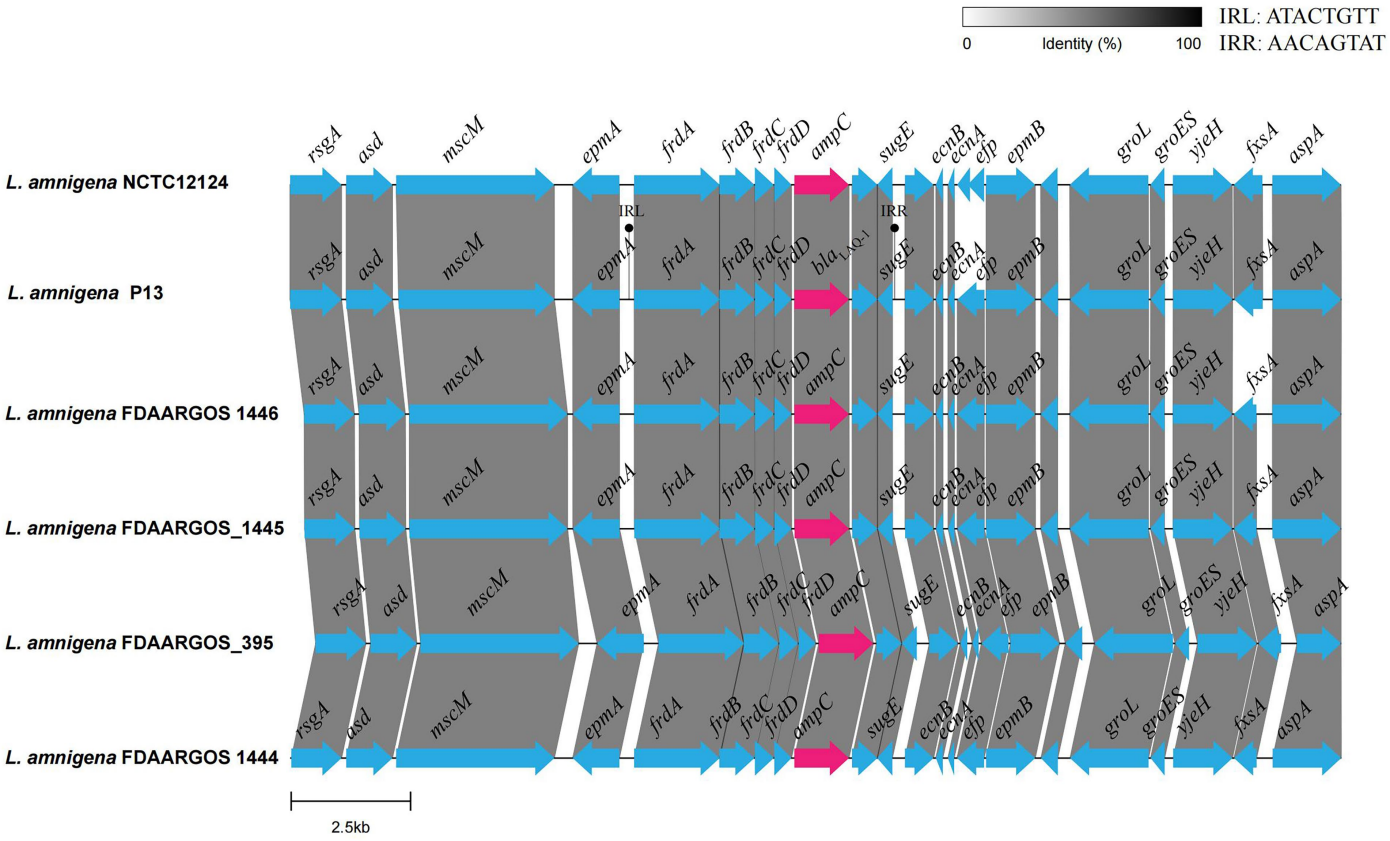
Comparative genomic analysis of the genetic context of *bla*_LAQ-1_ with the sequences carrying their homologous genes. The direction of genes is shown *via* an arrow. The *bla*_LAQ-1_ gene and putative *ampC* (*bla*_LAQ-1_-like) genes are colored red, and the other genes are colored blue. The predicted hypothetical genes are in gray. The sequences and their accession numbers are as follows: *L. amnigena* NCTC12124 (LR134135.1), *L. amnigena* FDAARGOS 1446 (CP077331.1), *L. amnigena* FDAARGOS_1445 (CP079896.1), *L. amnigena* FDAARGOS 1444 (CP077236.1) and *L. amnigena* FDAARGOS 395 (CP023529.1).

To analyze the genetic environment of *bla*_LAQ-1_ of *L. amnigena* P13 and its relatives, a fragment that was approximately 20 kb in length with the *bla*_LAQ-1_ gene at the center was queried against the NCBI nucleotide database. Five fragments of the highest similarities were retrieved, which were all from *L. amnigena* chromosomes (>99% coverage and >95.00% identity). Comparative genomic analysis of the six sequences (including the one from this work) showed that they shared conserved structures in both the gene context and the gene order, except that the gene context of *L. amnigena* NCTC12124 was slightly different from the other five (Figure 4). The *bla*_LAQ-1_ homologous genes identified in *L. amnigena* P13 and its relatives were located in the chromosomes, and no MGE was detected around them. This finding suggests that these genes may be intrinsic in the bacteria of this speice (Figure 4). On the other hand, although no MGE was predicted in the region surrounding *bla*_LAQ-1_, there is a pair of perfect 8 bp inverted repeats (IRs) on both sides of the fragment encoding *fraA-D*-*bla*_LAQ-1__−_*sugE*_,_ suggesting that they might play a role in mobilization and horizontal gene transfer (HGT) of this gene array.

### Comparative analysis of the plasmids carrying multiple resistance genes

In this study, 12 resistance genes related to different MGEs were identified in the plasmid pP13-67, including one quinolone resistance gene (*qnrS1*), two aminoglycoside resistance genes [*aph(6)-Id* and *aadA2*], two sulfonamide resistance genes (*sul1* and *sul2*), one β-lactam resistance gene (*bla*_TEM-1_), one truncated quaternary ammonium compound resistance gene (*qacEΔ1*), one diaminopyrimidine resistance gene (*dfrA12*), two copies of tetracycline resistance gene *tetA* and two copies of chloramphenicol/florfenicol resistance gene *floR*.

Comparative genomic analysis revealed that pP13-67 shared the highest identity with three plasmids, including p1 of *Klebsiella pneumoniae* JM45 (CP006657.1, 85.0% coverage and 99.94% identity), pEcl5-2 of *Enterobacter hormaechei* Eho-5 (CP047738.1, 70.0% coverage and 99.97% identity) and pN56639 of *Escherichia coli* CVM N56639 (CP043753.1, 73.0% coverage and 99.81% identity). The differences in the sequences of the four plasmids were mainly located in a ~22 kb multidrug resistance (MDR) region encoding a typical class 1 integron of pP13-67 (Figure 1).

Interestingly, this plasmid contained two identical fragments in a tandem repeat structure at positions 0–14 kb and 53–67 kb (from Tn*As1* to IS*903B*). The repeat sequence was composed of a number of MGEs (such as insertion sequences, ISs) and two resistance genes [*floR* and *tet(A)*], approximately 28 kb in length, which contributed to the high MIC levels for tetracycline (64 μg/mL) and florfenicol (128 μg/mL). Similar sequences were also found in the three plasmids mentioned above (with p1 of *Klebsiella pneumoniae* JM45 lacking the *floR* gene).

**Figure 5:**
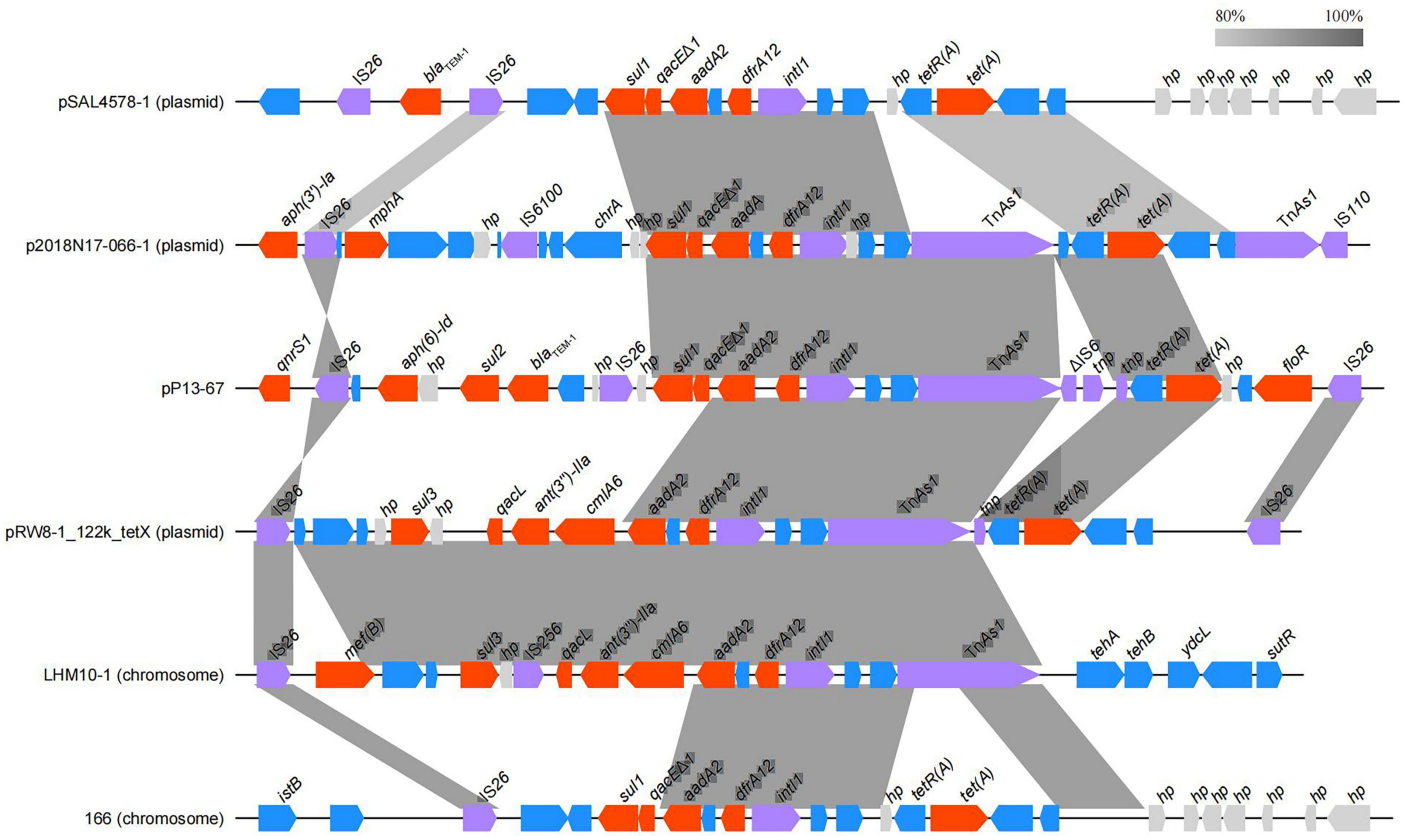
Comparison of the MDR region of pP13-67 with the related sequences on the other plasmids and chromosomes. Genes are denoted by arrows and colored according to gene function classification. Shading indicates the homologous regions.

To better characterize the distribution of the MDR-related sequences, the MDR region of pP13-67 was comparatively analyzed with other relative sequences. The results showed that the MDR region of pP13-67 shared the highest nucleotide sequence similarities with those of chromosomes or plasmids of bacteria of different species, including the chromosome of *Escherichia albertii* strain Sample 166 (CP070292.1, 88.0% coverage and 99.92% identity), the chromosome of *Escherichia coli* strain LHM10-1 (CP070292.1, 76.0% coverage and 99.97% identity), p2018N17–066-1 of *Klebsiella pneumoniae* strain 2018 N17-066 (CP044390.1, 86.0% coverage and 99.84% identity), pSAL4578-1 of *Salmonella enterica subsp*. enterica serovar 4,[5],12:i:-L-4578 (AP023310.1, 82.0% coverage and 99.97% identity), and pRW8-1_122k_tetX of *Escherichia coli* RW8-1 (MT219826.1, 79.0% coverage and 99.84% identity). Notably, there was a typical class 1 integron (organized with a 5-conserved segment [5-CS: *intI1*], a variable region [VR: *aad A2* and *dfrA12*]) and a 3-CS [3-CS: *qacE11*-*sul1*] on pP13-67 (Figure 5). The typical class 1 integron was also found in the remaining five sequences. The integron in pP13-67 was connected with transposon Tn*As1*, enabling its transfer between different positions in chromosomes and/or plasmids.

## Conclusion

In this study, on the basis of whole-genome sequencing, we identified a novel chromosome-encoded AmpC β-lactamase gene designated *bla*_LAQ-1_ in an environmental bacterium *L. amnigena* P13. Among all the reported functional β-lactamases, LAQ-1 shares the highest amino acid identity of 78.95% with ACT-22. *bla*_LAQ-1_ confers resistance to a number of β-lactam antimicrobials, including several first- to fourth-generation cephalosporins. In addition to the chromosomal *bla*_LAQ-1_, a number of resistance genes conferring resistance to tetracyclines, chloramphenicol, florfenicol and streptomycin were identified in the plasmid (designated pP13-67) of *L. amnigena* P13. All these findings will help in the elucidation of the resistance mechanisms of this unusual opportunistic pathogen and in the development of treatment methods for human (or animal) infections caused by *L. amnigena*.

## Data availability statement

The datasets presented in this study can be found in online repositories. The names of the repository/repositories and accession number(s) can be found at: https://www.ncbi.nlm.nih.gov/, CP099511.1, CP099512.1, MZ497396.1.

## Author contributions

QB, CC, XS, and DH: conceived and designed the experiments. AL, CY, LZ, SL, CF, LHZ, FD, and LW: performed the experiments. AL, YZ, JL JX, LZ, and DH: data analysis and interpretation. AL, CF, QB, CC, and DH: drafting of the manuscript. All authors contributed to the article and approved the submitted version.

## Funding

This study was supported by Zhejiang Provincial Natural Science Foundation of China (LGF19H200003); the Science & Technology Project of Taizhou City, China (21ywb126); the Science & Technology Project of Wenzhou City, China (N20210001); Technology Planning Project of Zhejiang Province (LGN19C180002) and the Science & Technology Project of Jinhua City, China (2022-4-017).

## Conflict of interest

The authors declare that the research was conducted in the absence of any commercial or financial relationships that could be construed as a potential conflict of interest.

## Publisher’s note

All claims expressed in this article are solely those of the authors and do not necessarily represent those of their affiliated organizations, or those of the publisher, the editors and the reviewers. Any product that may be evaluated in this article, or claim that may be made by its manufacturer, is not guaranteed or endorsed by the publisher.

## Footnotes

^1^ https://web.expasy.org/protparam/
