# Supplementary material for: Characterization and identification of a novel chromosomal class C β-lactamase, LAQ-1, and comparative genomic analysis of a multidrug resistance plasmid in Lelliottia amnigena P13
Source: Front Microbiol. 2022 Nov 23;13:990736. doi: 10.3389/fmicb.2022.990736 (PMC9727190; doi:10.3389/fmicb.2022.990736)
Supplement: Supplementary file 1 [file Table_1.doc]

**Table S1. Cloning primers for the blaLAQ-1 gene**

| Primer ^a^ | Sequence (5'-3')^b^ | Restriction endonuclease | Vector | Annealing temperature (℃) | Amplicon size (bp) |
| --- | --- | --- | --- | --- | --- |
| *orf-*blaLAQ-1-F | CGCGGATCCGACGACGACGACAAGCCTCAAACTGAAAAACAGATCGCTG | *BamH*I+DDDDK | pET 28a | 58 | 1,083 |
| *orf-*blaLAQ-1-R | CCCAAGCTTGGGTTATTTCAACGTGTCCAGGATACGATAC | *Hin*dIII |
| *pro-*blaLAQ-1-F | CGCGGATCCGCGTGCAAAATCCGGTGGTGGTGATC | *BamH*I | pUCP24 | 61 | 1,785 |
| *pro-*blaLAQ-1-R | CCCAAGCTTGGGATCGGTTATTTCAACGTGTCCAGGA | *Hin*dIII |

^a^Primers with “orf” were used to clone the ORF of the blaLAQ-1 gene, and primers with “pro” were used to clone the blaLAQ-1 gene with its promoter region;

^b^The underlined sequences represent the restriction endonuclease sites and their protective bases.
